# Magnetic resonance imaging findings and clinical manifestations in cerebral angiostrongyliasis from Dali, China

**DOI:** 10.1002/brb3.1361

**Published:** 2019-07-17

**Authors:** Bin Yang, Ling Yang, Yili Chen, Guangming Lu

**Affiliations:** ^1^ Department of Medical Imaging, Jinling Hospital Medical School of Nanjing University Nanjing China; ^2^ Department of Radiology Yunnan Cancer Hospital Yunnan China; ^3^ Department of Neurology People's Hospital of Dali Prefecture Dali China

**Keywords:** *Angiostrongylus cantonensis*, imaging diagnosis, magnetic resonance imaging, meningitis, tomography

## Abstract

**Purpose:**

To analyze magnetic resonance imaging (MRI) findings and clinical diagnosis and treatment data relating to *Angiostrongylus cantonensis* infection to gain insight into the disease.

**Materials and Methods:**

We retrospectively analyzed the epidemiology, clinical manifestations, diagnosis and treatment data, imaging manifestations, and outcomes of 27 patients who were clinically diagnosed with angiostrongyliasis and who underwent contrast‐enhanced brain MRI.

**Results:**

Patients with *A. cantonensis* infection had a history of eating raw mollusks in the endemic area, and they mainly presented with dizziness and headache of varying degrees and vomiting (*n* = 7). Laboratory examinations revealed increased peripheral blood and cerebrospinal fluid (CSF) eosinophils, as well as increased CSF protein levels. Brain MRI findings mainly included eosinophilic meningitis, whereas linear or nodular enhancement of the pia mater was observed in enhanced T1‐weighted and fluid‐attenuated inversion recovery images, accompanied by encephalitis or vasculitis. Meningitis manifested as multiple, thickened flow voids around the meninges, and contrast‐enhanced scans showed substantial enhancement in intracranial dilated and hyperplastic blood vessels.

**Conclusion:**

The possibility of *A. cantonensis* infection should be considered in the effective use of albendazole or mebendazole as a treatment. Combining clinical history with laboratory examination is helpful in diagnosing *A. cantonensis* infection. A final definite diagnosis can be confirmed by detecting larvae in the CSF. The administration of corticosteroids during pathogen therapy can substantially reduce the therapeutic response.

## 1 INTRODUCTION

Angiostrongyliasis is recognized as a zoonotic parasitic disease that is commonly caused by the ingestion of raw or undercooked gastropods containing the larvae of *Angiostrongylus cantonensis*, or vegetables contaminated by snails or slugs. Parasites migrate to the central nervous system (CNS) and can lead to the triad of eosinophilic meningitis (EM), encephalitis, and ocular angiostrongyliasis. The most common manifestation of angiostrongyliasis is EM, which is characterized by an increase in the number of eosinophils in the peripheral blood and cerebrospinal fluid (CSF). *Pomacea canaliculata* and *Erhaia jianouensis*, which are commonly found around endemic areas, are both naturally infected with angiostrongyliasis; additionally, people residing in these areas have a local custom of consuming snails and crustaceans (Wang, Shen, Li, Xue, & Wang, 2011). We collected and analyzed the imaging and clinical data of 27 patients with angiostrongyliasis and explored the magnetic resonance imaging (MRI) features of the disease to gain insight into this condition and its diagnosis.

## 2 MATERIALS AND METHODS

### 2.1 General information

The epidemiology, clinical manifestations, diagnosis and treatment data, outcomes, and imaging findings of 27 patients who were clinically diagnosed with angiostrongyliasis at the First Affiliated Hospital of Dali University between May 2014 and June 2017 were retrospectively studied. The patients included 12 men and 15 women, aged between 17 and 53 years, with a median age of 38 years. The study design was reviewed and approved by the Ethics Committee of Dali University.

### 2.2 Clinical diagnostic criteria

All patients had a history of ingesting raw or undercooked snails or shrimp and presented with symptoms 3 days to 1 week after ingestion. All patients shared similar symptoms, and disease onset was acute; all patients presented with dizziness and headache, six patients also had fever and chills, four patients experienced nausea and vomiting, two patients had muscle pain in the chest and back or lower limbs, and one patient presented with dysarthria. A marked increase in peripheral and CSF eosinophils was observed in 22 patients, ranging from 0.52 to 3.78 × 10^9^/L and from 10% to 83%, respectively. Increased CSF protein levels were observed in 21 patients, with an approximate range of 520–2,524 mg/L (Table 1). Sixteen patients were positive for *A. cantonensis* IgG in the CSF, and larvae were detected in the CSF in seven patients. In all patients, treatment with albendazole or mebendazole and corticosteroids was effective.

**Table 1:** Clinical manifestations of *Angiostrongylus cantonensis* infection from Dali, China (*n* = 27)

Clinical manifestations	Case number	Percentage
Headache	27	100
Low heat, fear of cold	6	22.2
Nausea and vomiting	6	22.2
Drowsiness	4	14.8
Pain in chest, back, or lower extremity muscles	2	7.4
Enunciation unclear	1	3.7
Mild meningeal stimulation sign	2	7.4

Note

The date, between May 2014 and June 2017.

### 2.3 Laboratory examination

Blood samples (K2‐ethylenediaminetetraacetic acid–anticoagulated venous blood) were collected from the median vein of the elbow in the morning and in the fasting state after admission. Routine blood tests (3 ml) were performed using a fully automatic blood cell analyzer (Mindray BC 6900; Mindray) and a microscope (Olympus). The blood samples were separated by serum (4 ml) after centrifugation, and the serum was subjected to biochemical tests using an automatic biochemical analyzer (Hitachi 7180; Hitachi). After centrifugation, the 3‐ml blood sample was separated from the bleeding serum, and antibodies against *A. cantonensis* were detected in the serum by using a micro‐enzyme‐linked immunosorbent assay (ELISA) using a young‐adult worm antigen with a molecular weight of 204 kDa, purified using a monoclonal antibody (Tsai et al., 2012).

The patients underwent lumbar puncture after admission, and 3 ml of CSF was collected for routine analysis, biochemical tests, cytology classification, specific antibody detection, and parasite detection. Specific antibody detection also involved ELISA detection reagents. The parasite was detected through centrifugation of the CSF and examined using a precipitation microscope (Olympus).

### 2.4 Examination methods

All patients underwent noncontrast and contrast‐enhanced brain MRI performed using a Toshiba 3.0‐T superconducting magnetic resonance scanner with a head and neck coil (Toshiba). The scanning sequences and parameters were as follows: routine fast spin echo (FSE) axial T1‐weighted imaging (T1WI; repetition time [TR] = 1,600 ms; echo time [TE] = 10 ms) and axial T2WI (TR = 4,000 ms; TE = 96 ms). Fluid‐attenuated inversion recovery (FLAIR) imaging of the corresponding sections was also performed, followed by FSE sagittal T2WI (TR = 4,071 ms; TE = 87 ms). Slice thickness was 6 mm, the interslice gap was 1 mm, and 20 slices were scanned for each sequence. The contrast agent administered for contrast‐enhanced MRI was gadolinium‐diethylenetriamine penta‐acetic acid (GD‐DTPA) at a dose of 0.1 mmol/kg. After intravenous injection of the contrast agent, axial, sagittal, and coronal T1WI scans were repeated, and an axial FLAIR sequence was performed as needed based on the patient's condition using the same slice parameters.

### 2.5 Image analysis

The MRI scans of the 27 patients were analyzed by two senior attending physicians with many years of experience in neuroimaging diagnosis. All observed imaging signs were recorded, and discrepancies in opinions were resolved through mutual discussion.

## 3 RESULTS

### 3.1 Epidemiological findings

The patients had the same epidemiological characteristics and resided in the same endemic area called Dali, China. *P. canaliculata* and *E. jianouensis* are common in this area, and people residing in that place have a local custom of consuming snails and crustaceans, which are commonly found around these areas, and are both naturally infected with angiostrongyliasis. They all had a history of eating aquatic plants, snails, broiler chickens, and shrimp. The intermediate host was *P. canaliculata*.

### 3.2 Clinical characteristics

The patients had similar clinical manifestations and acute onset of the disease, with symptoms appearing within 3 days to 1 week. All patients had varying degrees of dizziness and headache, which showed progressive aggravation. In addition to dizziness and headache (27/27), six patients had low fever (38–39°C) and chills (6/27), four patients experienced nausea and vomiting as well as lethargy (4/27), two patients had muscle pain in the chest, back, or lower limbs (2/27), one patient had unclear enunciation (1/27), and two patients had mild meningeal irritation (2/27) (Table 1).

### 3.3 Laboratory examination

**Figure 1:**
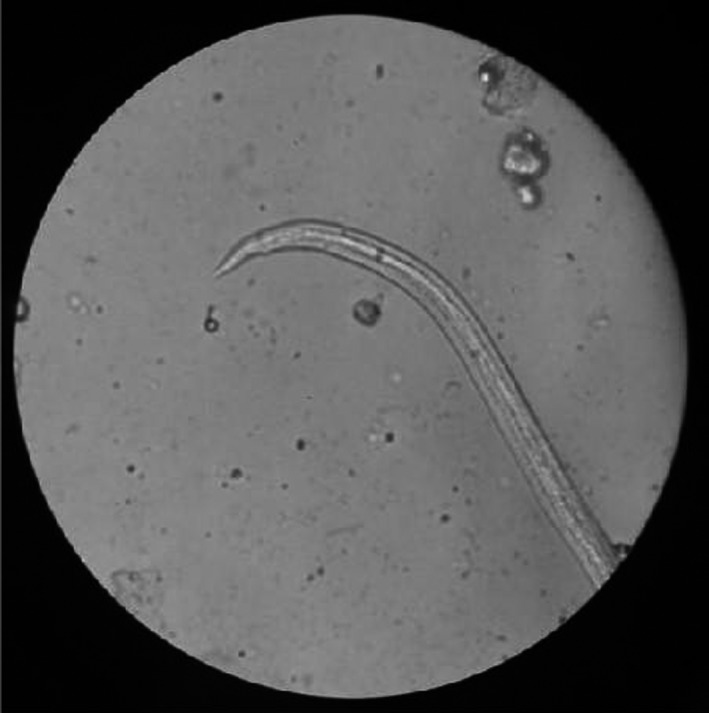
L3 larvae were imaged under phase‐contrast microscopy (×10). The worm body looks like a cotton thread, the tail is thinner, the surface skin is colorless, transparent, and smooth, and the body has a long strip of intestinal tube

Routine blood examination, blood biochemistry, routine CSF analysis, and biochemical and immunological CSF analyses were performed. No definite abnormalities were found in the blood biochemistry of patients: 17 patients had increased peripheral blood eosinophils (0.52–3.78) × 10^9^/L (normal [0.05–0.5] × 10^9^/L). The CSF was colorless and clear, and CSF eosinophil percentage was increased (by 10%–83% [normal <0.3%]) in all patients. In 21 patients, the total protein level in the CSF increased to 520–2,524 mg/L (normal 200–400 mg/L). CSF tested negative for bacteria, and glucose sugar and chlorine levels were normal in all patients. In 16 patients, the CSF was positive for anti‐*A. cantonensis* IgG, whereas larvae were detected in the CSF of seven patients (Table 2). We also detected larvae in the samples of *P. canaliculata* eaten by the patients (Figure 1).

**Table 2:** Laboratory examination of *Angiostrongylus cantonensis* infection from Dali, China (*n* = 27)

Laboratory examination	Case number	Percentage	Range
Elevated peripheral blood eosinophils	17	63.0	(0.52–3.78) × 10^9^/L
Normal peripheral blood eosinophils	10	37.0	(0.05–0.5) × 10^9^/L
Elevated CSF eosinophils	17	63.0	10%−83%
Normal CSF eosinophils	10	37.0	0%–0.3%
Elevated CSF protein	21	77.8	520–2,524 mg/L
Normal CSF protein	6	22.2	200–400 mg/L
Positive cytomegalovirus antibody IgG	8	29.6	+
Positive *Angiostrongylus cantonensis* antibody IgG	16	59.3	+
CSF positive for larvae	7	25.9	+

Note

The date, between May 2014 and June 2017.

Abbreviations: CSF, cerebrospinal fluid; IgG, immunoglobulin G.

### 3.4 MRI manifestations

**Figure 2:**
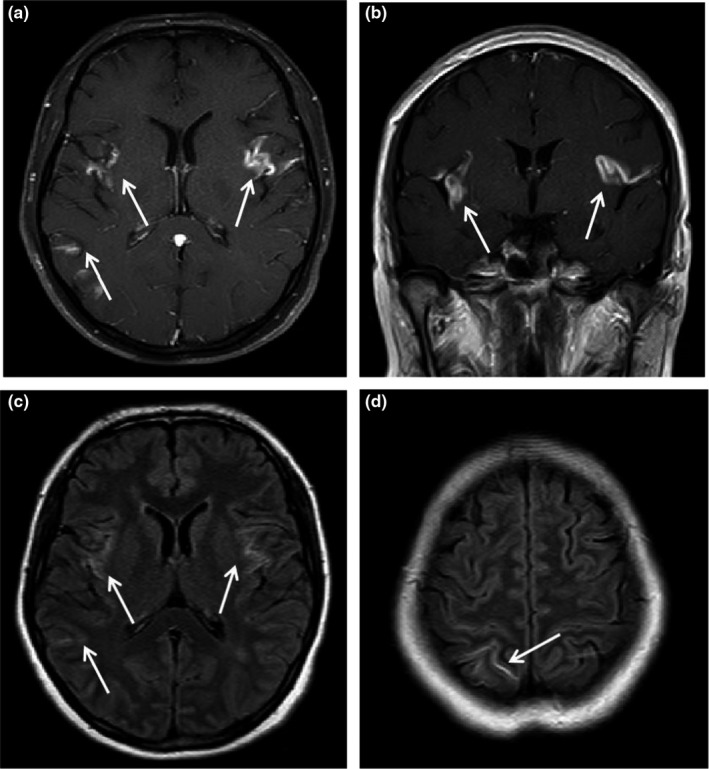
(a–d) A male, 35 years old. (a, b) Axial and coronal enhanced T1WI scans showing multiple long, abnormal enhancements in the pia matter. (c, d) Axial enhanced FLAIR image showing multiple long, abnormal enhancements in the pia matter, slightly lower than in the enhanced T1WI scan

**Figure 3:**
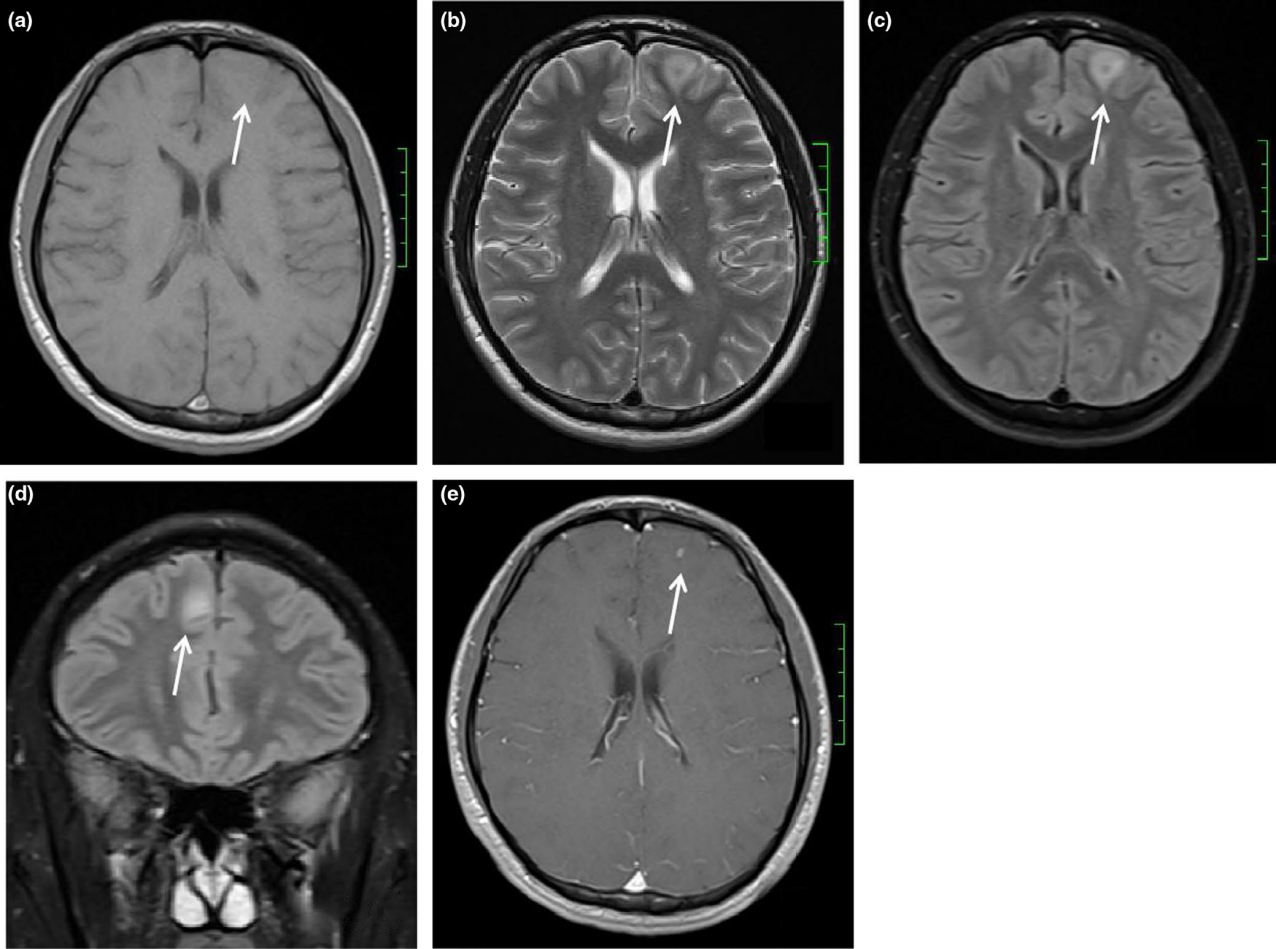
(a–d) Male, 46 years old. (a) T1 axis shows patchy iso‐signal in the left frontal lobe. (b) T2 axis shows patchy long T2 signal in the left frontal lobe. (c, d) Axis and coronal FLAIR show the bilateral frontal lobe lesions with high signal. (e) Axis enhanced shows the bilateral frontal lobe lesions with punctiform enhancement

The MRI examinations of all patients showed intracranial abnormalities (Table 3). The MRI findings showed thickening of the meninges alone in 16 patients, thickening of the meninges and intracranial nodules in four patients, intracranial nodules alone in another four patients, thickening of the meninges and perimeningeal vascular thickening in one patient, and perimeningeal vascular thickening alone in two patients. The specific manifestations are shown in Table 3. Meningitis mainly involved the pia mater, with no signal abnormalities observed in T1WI, T2WI, or FLAIR MRI. After the injection of GD‐DTPA, diffuse linear or nodular enhancements were observed in the pia mater in T1WI, without enhancement in the dura mater. Enhanced FLAIR imaging revealed mild‐to‐moderate enhancement in the lesioned meninges, which was slightly weaker than that observed in T1WI (Figure 2a–d). Encephalitis manifested as multiple patchy intracranial abnormalities in both gray matter and white matter, with diameters ranging from 4 to 12 mm. Lesions appeared as iso/hypointensities in T1WI and as hyperintensities in T2WI and FLAIR imaging. Nodular T1WI, T2WI, and FLAIR signals were observed in the lesion center. After the injection of GD‐DTPA, substantial nodular enhancement was observed in the lesion center (Figure 3a–e). Vasculitis manifested as multiple thickened flow voids around the meninges. After the injection of GD‐DTPA, substantial intracranial enhancement of increased thickening of blood vessels was observed, mostly around the meninges.

**Table 3:** Magnetic resonance features of *Angiostrongylus cantonensis* infection

MR diagnosis	Case number	T1WI	T2WI	FLAIR	Enhancement	Enhanced FLAIR
Simple meningitis	16	—	—	—	The pia mater shows marked linear/nodular enhancement	The pia mater shows mild‐to‐moderate linear/nodular enhancement
Meningitis with encephalitis	4	Multiple small patches of iso/hypointensities	Small areas of hyperintensity	Small areas of hyperintensity	Nodular enhancement and perichondrial enhancement	Enhancement of the pia mater
Simple encephalitis	4	Multiple iso/hypointensities	Hyperintense nodules	Hyperintense nodules	Nodular enhancement within the lesion	/
Meningitis with vasculitis	1	Flowing avoid effect of multiple blood vessels around the meninges	Multiple perimeningeal vascular thickening	Multiple perimeningeal vascular thickening	Nodular enhanced perichondrium and thickened blood vessels	The pia mater presents linear/nodular enhancement
Simple vasculitis	2	Flowing avoid effect of multiple blood vessels around the meninges	Multiple perimeningeal vascular thickening	Multiple perimeningeal vascular thickening	Clear enhancement of thickened vessels	Perimeningeal multiple thickening flow empty vascular

Note

— Indicates that there was no abnormality in the sequence; / Indicates that the sequence was not scanned.

Abbreviations: FLAIR, fluid‐attenuated inversion recovery; MR, magnetic resonance, T1WI, T1‐weighted imaging, T2WI, T2‐weighted imaging.

### 3.5 Treatment and outcomes

Relevant examinations were performed after the patients were hospitalized. Based on medical history, clinical manifestations, laboratory examinations, and brain MRI, various antiparasitic drugs, including albendazole and mebendazole, were successively administered for regular treatment (albendazole combined with dexamethasone), which accelerated the death of the parasite and shortened the duration of the disease. This was supplemented by anti‐infection, symptomatic support, and nutritional treatment. All patients were also treated with dexamethasone and prednisone, which can considerably reduce the side effects of treatment, inhibit the inflammatory reaction to the dead larvae, reduce the proportion of eosinophils, and alleviate headache. Albendazole (15–20 mg/kg/day) was administered as two doses, combined with dexamethasone (10 mg/day), for 9–27 days. This treatment resulted in the alleviation of headaches, and eosinophil levels returned to normal in routine blood and CSF analysis. Two patients presented with dry mouth, nausea, and other mild adverse reactions of the digestive system, which disappeared without treatment.

After being discharged from the hospital, partial patients returned irregularly for follow‐up brain MRI, which mainly showed a decrease in meningeal and nodular enhancement. After treatment, the process of larvae absorption was slower than the relief of clinical symptoms, but this eventually also returned to normal.

## 4 DISCUSSION

Angiostrongyliasis is caused by infection by stage III larvae of *A. cantonensis*. The larvae enter the human body via the gastrointestinal tract, where they penetrate the intestinal wall and circulate from the portal vein, inferior vena cava, right heart, and lungs to the left heart and then to the entire body via the arteries (Tseng et al., 2011). The parasites are neurotropic and preferentially infect the CNS. This primarily leads to EM and can cause death in severe cases. Granulomatous inflammation occurs in the CNS after the parasite dies. The adult parasite resides in the pulmonary arteries of rodents, whereas their larvae reside within the bodies of intermediate hosts, including a variety of mollusks, such as *Ampullaria crossean* (the most prevalent intermediate host), *Oncomelania* spp., snails, and shrimp (Wang, Wu, Wei, Owen, & Lun, 2012). The climate and geographical conditions of the Erhai Lake region in Yunnan, China, are highly suitable for the survival and reproduction of *A. crossean*. Hence, this species is widely distributed and highly adapted, and its natural *A. cantonensis* infection rate is 11.5% (Maretic et al., 2009). Residents around Erhai Lake commonly consume snails, and there is a high density of *A. crossean* around the highly populated towns in this region (Su et al., 2016). Direct ingestion of raw or undercooked mollusks and consumption of fresh vegetables contaminated with mollusk mucus are the most common routes of *A. cantonensis* infection in humans. Studies have shown that early intervention can enhance the therapeutic efficacy of drugs (He et al., 2011); therefore, early diagnosis is crucial for successful treatment.

The patients in this group were young and middle‐aged, with no statistically significant difference in the age of onset or sex, and no obvious specificity of clinical symptoms, which were less severe than those in infected children (Sawanyawisuth et al., 2013). The clinical manifestations of this disease included headache, delirium, fever, vomiting, and muscle pain in the chest and back or lower limbs, with meningeal irritation in a few patients. Severe headache was the most prevalent symptom, similar to previous case reports (Lv, Zhou, & Andrews, 2017; Tseng et al., 2011), which is mostly caused by the release of metabolites by the nematodes into the CNS, thereby leading to inflammatory reactions and elevating intracranial pressure (Tseng et al., 2011). The incubation period of this disease is generally between 1 day and 2 months, with an average of approximately 2 weeks. This is consistent with the time needed for the larvae to enter the CNS tissues and induce inflammatory reactions (Eamsobhana, 2014). The severity and incubation period of the disease may be related to the number of larvae consumed (Murphy & Johnson, 2013). Elevation of peripheral and CSF eosinophils (>10%) is a feature of angiostrongyliasis; eosinophils are critical regulators of cellular immune response, but only half of the patients in this study showed elevated eosinophils in the initial examination. In vitro culture studies (Gosnell & Kramer, 2013) have shown that, after parasitic infection, dramatic changes occur in the expression of cytokines and chemokines, thus promoting the production and activation of eosinophils, and infiltration of the meninges by lymphocytes, plasma cells, and eosinophils is common. The increase in CSF protein levels may be related to damage to the blood–brain barrier, but its underlying mechanisms remain unclear (Martins, Tanowitz, & Kazacos, 2015; Xie et al., 2017). In our patient group, 17/27 patients showed elevated peripheral and CSF eosinophil counts, whereas 21/27 patients showed elevated CSF protein levels, which have major implications for the diagnosis of this disease. In the CSF analysis, positivity for the anti‐*A. cantonensis* IgG antibody was highly suggestive of the disease (16/27 patients in this study). This facilitated a correct diagnosis, avoiding misdiagnosis or delayed diagnosis. Confirming a diagnosis of angiostrongyliasis involves detection of *A. cantonensis* larvae in the CSF or eyes (Yu et al., 2017); however, the detection rate is relatively low, which may easily lead to misdiagnosis of this disease. CSF was positive for the larvae in only 7/27 patients in this study. By combining this information with the epidemiological and medical history, a clinical diagnosis can be reached based on typical clinical symptoms, such as life history in the endemic area and a history of eating raw mollusks in the endemic area. Gradually increasing dizziness and headaches elevated peripheral and CSF eosinophils, and positive immunoassay results may also be useful (Xie et al., 2017). Human angiostrongyliasis is mostly a self‐limiting disease with low mortality.

Currently, most reports on *A. cantonensis* have focused on epidemiology, pathogenesis, and prevention and treatment, and few have summarized the clinical manifestations or imaging characteristics of sporadic cases of *A. cantonensis* (Andrade et al., 2018; Eamsobhana, 2010; Prasidthrathsint, Lewis, & Couturier, 2017). This study not only analyzed the clinical manifestations, but also analyzed and summarized the imaging characteristics and differential diagnosis of the disease, corresponding to its pathological basis. When the larvae of *A. cantonensis* reach the CNS, they reside in areas such as the pia mater, cerebrum, and cerebellum, where they release metabolites that lead to inflammation of the surrounding tissues; infiltration by large numbers of lymphocytes, eosinophils, and macrophages; local vasodilation; and gradual formation of eosinophilic granulomas around the parasites (Tsai, Shyu, Lim, Tyan, & Weng, 2016; Wang, Jung, Chen, Wang, & Li, 2015). Tsai et al. (2016) infected rabbit brains with *A. cantonensis* and reported widened intercellular spaces and infiltration of erythrocytes and inflammatory cells, indicating the occurrence of vasculitis after infection. The aforementioned changes constitute the pathological basis of MRI manifestations. The main MRI manifestations in this group were as follows: Meningeal lesions manifested as diffuse linear or nodular enhancements on enhanced T1WI and FLAIR imaging, and the degree of enhancement in FLAIR was slightly weaker than that in T1WI. Parenchymal lesions manifested as iso/hypointensities on T1WI and as hyperintensities on T2WI and FLAIR imaging. The central nodules were mostly parasites, and contrast‐enhanced scans of the parasitic region showed enhancements, whereas the surrounding granuloma did not. T1WI and T2WI scans of patients with vasculitis showed multiple thickened flow voids, and contrast‐enhanced scans showed considerable enhancement of hyperplastic and dilated blood vessels, mostly surrounding the meninges. Most of the patients in this study exhibited meningitis only on MRI, accompanied by encephalitis and vasculitis in some cases. This finding is similar to the manifestations of previously reported cases (Ouyang et al., 2012; Al Hammoud et al., 2016; Tsai, Chen, & Yen, 2013; Wang et al., 2010). In some patients, physical traces and microcavities are observed in the brain and spinal cord as a result of larval burrowing (Barratt et al., 2016), but this was not observed in our patient group. Shyu et al. (2014) infected rats with various numbers of larvae and reported widening of the subarachnoid space, ventricular enlargement, cerebrovascular dilation, and meningeal and parenchymal detachment. These changes became more severe as the number of larvae increased, and these rats had different prognoses.

Images of EM induced by angiostrongyliasis should be differentiated from the following diseases: (a) tuberculous meningitis: In patients with a history of tuberculosis, low fever, and afternoon tidal fever, other clinical manifestations such as meningeal extensive thickening, and radial or cast‐like strengthening more often involving the skull base and basement pool can be accompanied by meningeal, brain parenchymal miliary nodules, whereas serious cases can have tuberculoma, hydrocephalus, and cerebral infarction (Singh, 2017). (b) Cerebral gnathostomiasis: Both angiostrongyliasis and cerebral gnathostomiasis involve a history of ingesting raw food, share similar clinical symptoms, such as headache and dizziness, and show elevated CSF eosinophils. The manifestations of gnathostomiasis include multiple, diffuse intracranial hemorrhages and myelitis, multiple parenchymal hematomas, nontraumatic subdural hematoma, and unexplained subarachnoid hemorrhage (Kanpittaya et al., 2012), which can be used to differentiate it from angiostrongyliasis.

No recurrence of clinical symptoms or brain MRI findings occurred in the patients returned to hospital for review. Albendazole combined with dexamethasone was effective and may be a reliable drug for the treatment of *A. cantonensis*.

This study was limited by the small sample size. Larger samples of patients are needed in future studies. Additionally, assessing the intracranial recovery time was not possible because we were unable to obtain brain imaging findings throughout the convalescence period.

In conclusion, the patients had a history of residing in the endemic area, and the main clinical symptoms were gradually aggravated headache, dizziness, and the onset of disease, accompanied by nausea and vomiting, and an increased eosinophil count in the peripheral blood and CSF. The CSF was positive for anti‐*A. cantonensis* IgG, and the larvae could be detected in the CSF in some patients. The primary brain MRI manifestation of angiostrongyliasis was eosinophilic encephalitis, which was sometimes accompanied by meningitis or vasculitis. Albendazole combined with dexamethasone was effective as treatment for our patients. Combining patients' medical history with laboratory tests enables an accurate diagnosis. MRI provided some added value in facilitating the diagnosis of angiostrongyliasis.

## CONFLICT OF INTEREST

The authors declare they have no conflicts of interest according to the subject and matter of the present article.

## AUTHOR CONTRIBUTIONS

Bin Yang and Ling Yang conceived the idea of the study. Yili Chen performed the statistical analysis. Bin Yang and Ling Yang collected the data. Ling Yang and Yili Chen performed image analysis. Bin Yang and Ling Yang wrote the manuscript. Guangming Lu edited and reviewed the manuscript. All the authors discussed the results and commented on the manuscript.

## Data Availability

The data that support the findings of this study are available from the corresponding author upon reasonable request.
